# Food Preferences as a Positive Outcome for Adolescents with Type 1 Diabetes

**DOI:** 10.3390/nu17233752

**Published:** 2025-11-28

**Authors:** Grzegorz Sobek, Paweł Jagielski

**Affiliations:** 1 Faculty of Health Sciences and Psychology, Collegium Medicum, University of Rzeszów, 35-959 Rzeszów, Poland; 2 Department of Nutrition and Drug Research, Institute of Public Health, Faculty of Health Sciences, Jagiellonian University Medical College, 31-066 Krakow, Poland; paweljan.jagielski@uj.edu.pl

**Keywords:** adolescents, type 1 diabetes, food preferences, taste perception

## Abstract

Background/Objectives: Sensory properties of food, such as taste and smell, are the main factors influencing the preference or rejection of a given food product, especially among children. The aim of this study was to compare the food preferences of teenagers with diabetes to those of a group of healthy children. Additionally, we assessed the influence of children’s taste perception on food preferences. Methods: The study involved 102 adolescents with type 1 diabetes aged 11–15, including 55 girls and 47 boys. We used a questionnaire that consisted of 63 photos of various food products, dishes, and drinks. For the taste evaluation, we used paper strips impregnated with four basic tastes (sweet, sour, bitter, and salty). Results: Healthy teenagers were more likely to self-report preferences for sweet foods. The overall preference score for sweet products included in the study was higher in the control group. The median score for this group was 4.21 (3.92–4.42), and for the diabetes group, the median score was 4.03 (3.68–4.26) (*p* = 0.0008). Preferences for bitter-tasting vegetables and fruits were clearly higher in teenagers with type 1 diabetes. The overall preference score for bitter taste products included in the study was higher in the diabetes group. The median score for this group was 3.71 (3.00–4.14), and for the control group, the median score was 2.86 (2.14–3.50) (*p* < 0.0001). Conclusions: Adolescents with diabetes showed greater acceptance of certain bitter-tasting foods compared to their healthy peers. Lower preferences for sweet and higher preferences for bitter foods can be taken into account in establishing nutritional plans for adolescents with type 1 diabetes.

## 1. Introduction

Diabetes mellitus is a group of metabolic diseases characterized by hyperglycemia resulting from the defective secretion and/or action of insulin. Chronic hyperglycemia is associated with damage, dysfunction, and failure of various organs, in particular the eyes, kidneys, nerves, heart, and blood vessels [1]. Type 1 diabetes (T1DM) is one of the most common chronic diseases of childhood [2]. Currently in Poland, according to estimates, there are around 180,000 patients with type 1 diabetes, including several thousand in the age of 0–19 [3]. The goals of T1DM management include maintaining normal growth and development, achieving blood glucose control, maintaining optimal nutritional status, and preventing complications. Insulin replacement is the mainstay of treatment for T1DM. However, optimal therapy also requires a careful balance of food, insulin, and physical activity [4]. Dietary guidelines for children with type 1 diabetes are similar to those for the general population, and nutrition education for families of children with type 1 diabetes includes recommendations for overall healthy eating and efforts to achieve and maintain optimal weight for height. Patient education initiatives in Poland focus on providing comprehensive nutritional guidance, including carbohydrate counting, portion control, meal planning, and making healthier food choices. In the last five years, many options have been introduced, ranging from online diet plans available on the National Health Fund website to telemedicine consultations organized by the national nutrition education center [5]. However, the available literature has identified many young people with T1DM who did not adhere to dietary recommendations and did not follow the dietary guidelines for their disease [6,7].

Food preferences, particularly in children, are one of the most important factors determining food choices [8]. Food preferences change throughout life and are influenced by patterns acquired in childhood, cultural and social environments, personality traits that shape our attitudes towards new foods, previous consumer experiences, fashion, and advertising [9]. The level of nutritional knowledge of children and their parents is a factor that influences dietary choices and compliance with dietary recommendations. Additionally, it may affect taste and nutritional preferences, which are modifiable, especially over a longer period of time [10]. Sensory properties of foods are major factors influencing food acceptance and rejection, particularly in children. Food perception play a critical role in shaping appetite control and eating behavior, influencing food choices and energy intake [11]. A reduced level of sensory sensitivity is a factor that should be considered when analyzing food choices. Several publications have confirmed taste disturbances in adults with type 2 diabetes (T2DM) [12,13,14] and type 1 diabetes (T1DM) [15,16]. Few studies suggest such a relationship in the group of children and adolescents with type 1 diabetes [17,18].

The first part of the results of this study has been published [19], and the results in this publication looked at a detailed assessment of taste perception and olfactory function in adolescents with type 1 diabetes. The interdependence between food preferences and taste sensitivity was most often examined in individuals with type 2 diabetes in the context of food consumption, excessive body weight and obesity. For patients with type 1 diabetes, achieving and maintaining optimal glycemic control is crucial for overall health. Consequently, a thorough understanding of the mechanisms and factors influencing dietary choices, especially in relation to foods rich in simple carbohydrates among young patients with type 1 diabetes, is therefore very important. This study aims to compare the food preferences of children with type 1 diabetes to those of healthy children. Additionally, we will analyze the influence of taste sensitivity on the nutritional preferences of the subjects.

## 2. Materials and Methods

The study enrolled 148 adolescent patients with Type 1 Diabetes (T1DM) who regularly attend the specialist diabetes clinic for children at the Provincial Clinical Hospital in Rzeszów. We included 102 of them in our analysis, as they completed a food preference questionnaire.

Inclusion criteria comprised diagnosis of T1DM, intensive insulin therapy, an age range of 11 to 15 years, and a diabetes duration of at least 12 months. The exclusion criteria included type 2 diabetes and syndromic diabetes. We further excluded patients with acute or chronic disease interfering with taste function and those receiving medications known to affect olfaction and gustation.

A control group, comprising 100 healthy adolescents matched for age (11–15 years), was selected from a local school in the Podkarpackie Voivodeship, Poland. Both the diabetic and control groups were assessed for taste sensitivity and food preferences as described below. Participants were asked to refrain from eating, drinking (except room-temperature water), brushing their teeth, and chewing gum for 1 h before the examination. Parents of children from the research and control group gave written consent to participate in the study. The study was conducted following the ethical standards set out in the relevant version of the Declaration of Helsinki and Polish national regulations. The study was approved by the Institutional Bioethics Committee of the University of Rzeszów on 14 March 2019 (resolution no. 09/03/2019) and all relevant administrative bodies.

### 2.1. Assessment of Taste Function

Gustatory assessment was performed using the “Taste strips” from Burghart Messtechnik. The test used in clinical practice consisted of 16 taste-impregnated filter papers. Each of the 16 taste stimuli was impregnated with one of these four tastes: sweet, sour, salty, and bitter. Four concentrations of each taste quality were used: sweet (0.05, 0.1, 0.2, and 0.4 g/mL sucrose), sour (0.05, 0.09, 0.165, and 0.3 g/mL citric acid), bitter (0.0004, 0.0009, 0.0024, and 0.006 g/mL quinine hydrochloride) and salty (0.016, 0.04, 0.1, and 0.25 g/mL sodium chloride). To assess sensitivity, “Taste Strips” were placed on the middle of their tongue. Participants were then asked to close their mouths and indicate whether the perceived taste was sweet, sour, salty, bitter, or tasteless. The taste strips were presented in a randomized order, starting with the lowest concentration. Each correct answer was granted 1 point (maximum 4 points for each taste and 16 points for the whole test score). Before starting the test, 4 basic taste qualities were explained using food products.

### 2.2. Food and Beverage Preference Questionnaire

Food preferences were measured using a suitable assessment in a children’s dietary questionnaire based on a previous research questionnaire proposed by Jilani et al. [20]. The questionnaire consisted of 63 photos of various food products, dishes, and drinks (e.g., cornflakes, lettuce, bananas, French fries, kebab, fruit jam, chocolate, milk, cola). Children and adolescents were asked to indicate their likes on a 5-point facial hedonic scale, ranging from 1 meaning “do not like at all” to 5 meaning “like very much”. Respondents could indicate whether they did not know or had never tried a given food product.

### 2.3. Taste Preference Score

Cumulative preference scores for sweet, fatty, salty, and bitter tastes were calculated from 41 selected items (foods) from the Food and Beverage Questionnaire. To define the assessed food products according to their taste, a preliminary survey was conducted. Respondents were asked to classify individual food products into five tastes (sweet, bitter, salty, sour, and fatty). Only items that were assigned identically by all evaluators were taken into account for taste score calculations. This means we excluded foods that were associated with more than one taste. In addition, the items in which they were granted “I’ve never tried/I don’t know” responses were also excluded. No products were classified as having a sour taste. The taste preference score was calculated as the arithmetic mean of all results of items assigned to a given taste quality category. For all the taste quality groups, the alpha Cronbach was ≥0.60, supporting internal reliability.

#### Sociodemographic and Anthropometric Data

In addition, data regarding age, sex, weight, and height were collected. In the case of healthy children and adolescents, the data were obtained from the school nurse, and in children and adolescents with T1D, from the patient’s records. The body mass index (BMI) was calculated based on the weight and height [21], which was subsequently interpreted based on the national BMI percentile charts (OLAF and OLA) [22] for female and male participants aged from 11 to 15 years.

### 2.4. Statistical Analysis

Descriptive statistics were calculated: mean, standard deviation, median, and the first and third quartiles (Q1–Q3). Compliance with the normal distribution of quantitative variables was checked using the Shapiro–Wilk test. In order to check the differences between the two groups (boys and girls) for the analyzed quantitative or ordinal variables, the Mann–Whitney U-test was used, and chi-squared test for qualitative variables. Statistical analyses were performed using PS IMAGO PRO 10 (IBM SPSS Statistics 29, Armonk, NY, USA). The level of statistical significance was set at *p* < 0.05. To minimize false positives, a Bonferroni correction for multiple comparisons was applied. 0.05 was divided by the number of tests, which is 61, resulting in a value of 0.0008. In this study, results were considered satisfactorily significant when the *p*-value was ≤0.0008.

## 3. Results

### 3.1. Characteristic Study Group and Control Group

Boys constituted 52% of all study participants. The average age of respondents in both groups was approximately 13 years. Both groups of teenagers were similar in age. The average value of the subjects’ BMI was 20.0, with no significant differences between groups. There were also no significant differences between the groups based on gender. In children and adolescents with type 1 diabetes, the average duration of diabetes was approximately 6 years (mean ± SD: 6.39 ± 3.37). The minimum duration of diabetes was one year, and the maximum duration of the disease was 14 years. The characteristics of this sample are shown in Table 1.

### 3.2. Taste Recognition

**Figure 1:**
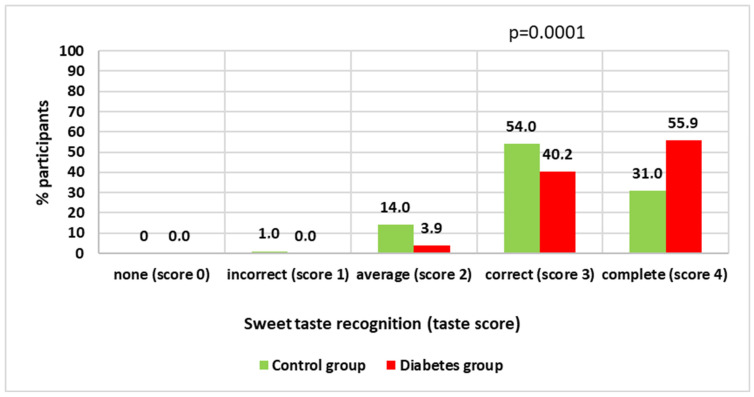
The overall evaluation of the recognition of sweet taste.

Our results of the study on the recognition of four basic tastes showed that the overall scores of adolescents with T1DM and healthy adolescents are similar. In particular, the sour, salty, and bitter taste recognition scores of adolescents with diabetes were consistent with those of control children. Mean scores for the individual taste qualities were in T1DM and control group as follows: sour taste (mean ± SD: 2.32 ± 0.86 and mean ± SD: 2.40 ± 0.86, *p* = 0.4383), salty taste (mean ± SD: 2.83 ± 1.06 and mean ± SD: 2.92 ± 0.98, *p* = 0.6223), and bitter taste (mean ± SD: 2.84 ± 1.06 and mean ± SD: 2.94 ± 1.01, *p* = 0.5389). However, there was a significant difference between the groups in the sweet taste test. The mean score in the diabetic group was (mean ± SD: 3.48 ± 0.60), and in the control group, it was (mean ± SD: 3.15 ± 0.69), (*p* < 0.0001). More than half, 55.9% of teenagers with diabetes, correctly identified all four intensities of sweet taste. Much fewer, 31%, of healthy children passed the test without any mistakes. Only 3.9% of diabetics had greater problems with recognizing the taste of sweet compared to 14.0% of healthy teenagers [Figure 1]. This means that they recognized one or two qualities of sweet taste (average and incorrect recognition). All tested teenagers correctly recognized the strongest of the four sweet taste qualities. Detailed results of the adolescents’ sour, salty, and bitter taste recognition are available in the Supplementary Materials [Figures S1–S3].

### 3.3. Food and Beverage Preference

Healthy teenagers were more likely to self-report preferences for sweet foods and drinks. In teenagers from the control group, the average scores for preferences for certain products associated with sweet taste were higher. In the case of chocolate flakes, the mean score in the control group was (mean ± SD: 3.92 ± 1.06), and in the T1DM group, it was (mean ± SD: 3.47 ± 1.28). Similarly, higher values were obtained for the control group for chocolate pudding; the mean score for the control group was (mean ± SD: 3.51 ± 1.22) and for the T1DM group was (mean ± SD: 2.94 ± 1.33), those for donuts were (mean ± SD: 4.70 ± 0.63) and (mean ± SD: 4.36 ± 0.69), those for fruit jam were (mean ± SD: 4.70 ± 0.46) and (mean ± SD: 4.41 ± 0.59), those for croissant with chocolate were (mean ± SD: 3.68 ± 1.30) and (mean ± SD: 3.32 ± 1.19), those for chocolates were (mean ± SD: 4.78 ± 0.49) and (mean ± SD: 4.47 ± 0.70), those for cookies were (mean ± SD: 4.11 ± 1.01) and (mean ± SD: 3.59 ± 1.18) and those for ice tea were (mean ± SD: 4.06 ± 1.09) and (mean ± SD: 3.68 ± 1.26), respectively. All these items met the initial significance requirement of *p* < 0.05. The restrictive significance level of ≤0.0008 adopted in the study showed differences only in the case of donuts. The overall preference score for sweet products included in the study was significantly higher in the control group (median ± Me = 4.21 (3.92–4.42) than in the T1DM group (median ± Me = 4.03 (3.68–4.26), *p* = 0.0008) (Table 2).Preferences for bitter-tasting vegetables and fruits were clearly higher in teenagers with type 1 diabetes. Higher scores were shown for most items, i.e., broccoli (mean ± SD: 3.69 ± 1.04) compared to (mean ± SD: 2.38 ± 1.30) in the control group, as well as red cabbage ((mean ± SD: 3.97 ± 0.99) and (mean ± SD: 3.12 ± 1.18)), brussels sprouts ((mean ± SD: 2.75 ± 1.22) and (mean ± SD: 1.78 ± 1.04)), and spinach ((mean ± SD: 3.00 ± 1.23) and (mean ± SD: 2.80 ± 1.36)), respectively. The overall preference score for bitter taste products included in the study was significantly higher in the diabetes group (median ± Me = 3.71 (3.00–4.14) than in the control group (median ± Me = 2.86 (2.14–3.50) (*p* < 0.0001) (Table 3). For salty and fatty taste, no significant differences in preference scores were found between the study groups. Data are available in the Supplementary Materials (Tables S1 and S2).

### 3.4. Sweet, Bitter and Salty Taste Food and Beverage Score Depending on the Results of Taste Strips Test

Teenagers in the control group are more likely to prefer sweet products compared to those in the diabetes group. However there were no differences in the results of sweet taste preference due to the results achieved in the taste strips test (Table 4). Teenagers from the diabetic group with high scores on the bitter taste test (correct and complete) are significantly more likely to prefer bitter products. The scores among those with a score of 3 for the diabetic group are (median ± Me = 3.71 (3.21–4.14)) compared to the control group (median ± Me = 3.00 (2.14–3.57) (*p* < 0.0001)), and among those with a score of 4 for the diabetic group are (median ± Me = 3.86 (3.43–4.29)) compared to the control group (median ± Me = 3.00 (2.29–3.57) (*p* < 0.0001)) (Table 5). No significant differences in salty food preference scores were observed between the diabetic and control groups based on taste test results.

## 4. Discussion

No singular, universally applicable dietary regimen exists for all individuals with diabetes. However, it is known that patients with type 1 diabetes should follow dietary recommendations consistent with the general population’s dietary guidelines [7,23]. However, we can agree that people with type 1 diabetes should limit their intake of easily digestible carbohydrates and adhere to a well-balanced diet. Insulin therapy should be tailored to the patient’s eating habits, meal composition (including carbohydrate, protein, and fat content), lifestyle, and physical activity level. In addition, individualized meal planning should focus on personal preferences, needs, and goals [24]. Taste preferences and liking are recognized as key determinants of food choice [25]. Certain taste preferences are innate, such as a preference for sweet and salty flavors, and a dislike for bitter and sour tastes [26]. What is important is that taste preferences are subject to modification through the learning and unlearning process [27]. We anticipated that, despite potential differences in the typical daily diet of adolescents with and without diabetes, sweet taste preferences would remain similar. Conversely, the results indicate that hedonic ratings for sweet foods, expressed as a sweet preference score, are lower among teenagers with type 1 diabetes when compared to their healthy counterparts. This diminished preference for sweet foods, as self-reported, is a particularly interesting observation because it concerns a group in which the content of carbohydrates, especially simple sugars, is of particular importance for glycemic control and maintaining health. It is well-established that individuals with diabetes are advised to restrict the consumption of snacks high in simple sugars. Previous studies tested the hypothesis that diabetic patients have a greater liking for certain types of taste stimuli (e.g., sweet and salty), which contributes to worsened glycemic control. In some studies involving type 2 diabetes, it was partially confirmed and associated with different abilities to perceive taste stimuli [28,29]. Although other research does not corroborate differences in the perception of sweet taste sensations [17], one recent study showed that children and adolescents with T1DM significantly more often correctly recognized sweet taste compared to healthy children [30]. Our own research conducted on a larger group of young patients confirmed their high sensitivity to sweet taste [19]. We therefore concluded that higher sensitivity to sweet taste in young diabetics is associated with a lower preference for sweet foods. Most studies confirm that individuals with a high sweetness threshold tend to prefer foods with higher sugar content [31]. However, a more detailed comparison of food preferences across varying degrees of sweet taste sensitivity (none, incorrect, average, correct, complete) in both study groups indicates that sweet food preferences do not depend on the level of sweet taste sensitivity. Therefore, we believe that concluding food preferences based on taste sensitivity assessments may not be sufficient.

Many studies have indicated that food preference is a complex phenomenon, grounded in various physiological, psychological, and cultural factors [32,33,34]. The food preferences and choices of a population are additionally linked to economic variables such as income [35]. Numerous social/environmental factors, including social norms, early food experiences, availability, parental preferences, and nutrition knowledge, can also influence children’s food preferences [36,37,38].

An increasing number of studies indicate a lower perception of tastes and smells among adults with type 1 diabetes [39,40,41,42]. One recent study involving adult patients with type 1 diabetes found lower perception of sour, bitter, and salty tastes compared to the control group, while sweet taste perception was at a similar level [43]. As we mentioned in our study, some differences in test results were observed only for the sweet taste. It is thought that if taste and taste perception are impaired, this may affect dietary adherence and food choices.

In the case of children and adolescents, the few results of taste perception tests cited above exhibit differences. These stem from variations in methodologies and the limited size of research cohorts. While a link between sweet food preference and consumption has been noted, it is possible that taste preference alone does not fully account for sweet product intake. Other factors, such as food’s smell or texture [44], or broader behavioral considerations like health or weight concerns, may also influence food choices and daily consumption [45]. In adults, reduced taste sensitivity or olfactory impairment often results from diabetic neuropathy, hyperglycemia, oral diseases, or medications commonly prescribed for diabetes [46,47,48]. Impaired taste perception in T1DM is associated with the duration of the disease and complications, especially peripheral neuropathy [12]. Therefore, we concluded that an additional aspect that should be considered in sensory examinations, especially in children with diabetes, is the duration of diabetes.

Our results of taste perception in children with type 1 diabetes did not show differences in sensitivity to bitter taste, although some previous studies suggested such a relationship [17,18]. It was also noticed that children and adolescents with type 1 diabetes seemed to prefer fruit and vegetables more than the control group. In our analysis, when comparing the preference scores among people who have a similar, high level of bitter taste sensitivity (correct and full recognition), we noticed a difference between the diabetics and the control group. Diabetics showed significantly higher levels of preferences for bitter products, which in the study were represented mainly by vegetables. Some previous studies have shown that young patients with type 1 diabetes had a greater preference for bitter or sour foods. This was attributed to an impaired ability to recognize these stimuli. Our study results [19] suggest that such differences in perception apply to young patients with a disease duration exceeding 10 years, as this increases the likelihood of developing diabetic complications, such as neurological disorders. In the study group, the average duration of diabetes was approximately 6 years. Therefore, we believe that the differences in preference scores between the study groups must be due to other factors that we did not consider in our research.

We certainly need to take into account possible reporting errors by diabetics. Young patients with chronic diseases are particularly likely to provide health-promoting responses. Another possible explanation for altered preferences is the increased motivation among children with diabetes to eat healthier meals. They showed a preference for food categories often avoided by many teenagers, such as certain fruits and especially vegetables, which, due to their chemical composition, tend to have a bitter, astringent, or sour taste [49]. It should also be added that adolescents with type 1 diabetes are regularly educated during visits to the diabetes clinic to improve their diet (e.g., increasing the consumption of fruit and vegetables or limiting sweets) as part of the treatment of the disease to maintain good metabolic control. This may have led our participants to prefer and perhaps consume these food categories to achieve greater compliance with diet-related diabetes management recommendations. In contrast, healthy children usually consume a full range of products and do not control the number of sweets in their diet. Some studies show that children with type 1 diabetes consume significantly fewer carbohydrates than their peers [50,51].

In summary, altered food preferences observed in adolescents with type 1 diabetes may be a consequence of the disease itself or behaviors adopted as a part of the diabetic lifestyle. Some studies show that individuals with type 1 diabetes demonstrate healthier eating habits than healthy peers [52,53]. However, these altered preferences may also indicate a more restrictive approach to setting dietary standards for adolescents with type 1 diabetes. This may include the appropriate selection of sugars or avoiding sweet snacks between main meals. Another important finding may be that a higher acceptance of bitter foods, especially vegetables high in fiber, allows them to be included more often in the diet of young type 1 diabetes. Numerous clinical studies highlight the beneficial effects of a regular diet rich in fiber and limited in carbohydrates on metabolic control of diabetes [54,55,56].

Our results should be interpreted in light of the limitations of the study. Firstly, the results cannot be generalized to all children and adolescents affected by T1D, given that the study cohort comprised a convenience sample recruited from a single pediatric diabetes center. Secondly, we did not take into account various factors influencing food preferences, such as economic status, physical activity, adolescent personality, or motivation. A causal interpretation of the observed associations should not be performed due to the cross-sectional design used. Despite the limitations, our study is one of the few conducted on a fairly large, age-homogeneous group and may contribute to a better understanding of diet-related relationships in the population of people with type 1 diabetes. The study used multiple comparison correction, which significantly reduces the risk of false positive results.

## 5. Conclusions

Our data suggests that there are differences in food preferences between adolescents with type 1 diabetes and their healthy peers, particularly regarding preferences for sweet and bitter foods. Young diabetic patients showed a higher acceptance of certain foods typically characterized by a bitter taste compared to their healthy peers. We also found that there is a significant association between greater acceptance of bitter foods and better bitter taste perception in young diabetics. Further research is needed to understand the differences in dietary preferences among adolescents with diabetes, as such insights may be useful in shaping the dietary strategies for young patients with type 1 diabetes.

## Figures and Tables

**Table 1:** Characteristics of the study and control group.

		Diabetes Group *n* = 102	Control Group *n* = 100	*p*-Value
female adolescents [%] male adolescents [%]		55 47	45 55	0.2048 ^a^
Age	M ± SD Min-Max	12.86 ± 1.76 10.0–15.0	12.99 ± 1.40 10.0–15.0	0.7289 ^b^
BMI [kg/m^2^]	M ± SD Min-Max	20.04 ± 3.14 15.3–31.0	20.14 ± 4.91 14.5–39.2	0.1911 ^b^

^a^ cχ^2^ test. ^b^ U Mann–Whitney test.

**Table 2:** Sweet food and beverage taste preference score.

	Diabetes Group					Control Group					*p*-Value
	Mean	SD	Median	Min.	Max.	Mean	SD	Median	Min.	Max.	
sweet preference score	3.99	0.44	4.03	3.05	4.84	4.17	0.57	4.21	0.00	5.00	0.0008 ^a^
banana	4.18	0.94	4.00	1.00	5.00	4.24	1.04	5.00	1.00	5.00	0.3332 ^a^
lemonade	4.11	0.97	4.00	1.00	5.00	4.24	1.01	5.00	1.00	5.00	0.2193 ^a^
Coca Cola	4.19	0.91	4.00	1.00	5.00	4.35	0.99	5.00	1.00	5.00	0.0801 ^a^
Coca Cola light	3.63	1.23	4.00	1.00	5.00	3.90	1.22	4.00	1.00	5.00	0.0933 ^a^
cornflakes	4.26	0.81	4.00	1.00	5.00	4.19	0.85	4.00	1.00	5.00	0.6020 ^a^
chocolate flakes	3.47	1.28	4.00	1.00	5.00	3.92	1.06	4.00	1.00	5.00	0.0189 ^a^
jelly candy	3.85	1.22	4.00	1.00	5.00	3.72	1.33	4.00	1.00	5.00	0.5411 ^a^
milk chocolate	4.35	0.68	4.00	2.00	5.00	4.43	0.76	5.00	2.00	5.00	0.2656 ^a^
fruit yogurt	3.65	1.21	4.00	1.00	5.00	3.60	1.32	4.00	1.00	5.00	0.9642 ^a^
chocolate pudding	2.94	1.33	3.00	1.00	5.00	3.51	1.22	4.00	1.00	5.00	0.0023 ^a^
donuts	4.36	0.69	4.00	2.00	5.00	4.70	0.63	5.00	1.00	5.00	0.0004 ^a^
Ice cream	4.64	0.74	5.00	1.00	5.00	4.81	0.47	5.00	2.00	5.00	0.2958 ^a^
ice tee	3.68	1.26	4.00	1.00	5.00	4.06	1.09	4.00	1.00	5.00	0.0316 ^a^
fruit jam	4.41	0.59	4.00	3.00	5.00	4.70	0.46	5.00	4.00	5.00	0.0021 ^a^
honey	4.04	0.89	4.00	1.00	5.00	4.23	0.78	4.00	2.00	5.00	0.1463 ^a^
croissant with chocolate	3.32	1.19	3.00	1.00	5.00	3.68	1.30	4.00	1.00	5.00	0.0218 ^a^
chocolates	4.47	0.70	5.00	2.00	5.00	4.78	0.49	5.00	3.00	5.00	0.0030 ^a^
cake	3.59	1.18	4.00	1.00	5.00	4.11	1.01	4.00	1.00	5.00	0.0019 ^a^
fruit juice	4.68	0.53	5.00	3.00	5.00	4.85	0.36	5.00	4.00	5.00	0.0872 ^a^

^a^ U Mann–Whitney test.

**Table 3:** Bitter food and beverage taste preference score.

	Diabetes Group					Control Group					*p*-Value
	Mean	SD	Median	Min.	Max.	Mean	SD	Median	Min.	Max.	
bitter preference score	3.54	0.76	3.71	1.57	5.00	2.85	0.86	2.86	1.14	4.57	<0.0001 ^a^
broccoli	3.69	1.04	4.00	1.00	5.00	2.38	1.30	2.00	1.00	5.00	<0.0001 ^a^
red cabbage	3.97	0.99	4.00	1.00	5.00	3.12	1.18	3.00	1.00	5.00	<0.0001 ^a^
brussels sprout	2.75	1.22	3.00	1.00	5.00	1.78	1.04	1.00	1.00	5.00	<0.0001 ^a^
spinach	3.00	1.23	3.00	1.00	5.00	2.80	1.36	3.00	1.00	5.00	0.3881 ^a^
asparagus	3.31	1.26	3.00	1.00	5.00	2.86	1.34	3.00	1.00	5.00	0.0225 ^a^
lettuce	3.96	1.09	4.00	1.00	5.00	3.52	1.33	4.00	1.00	5.00	0.0247 ^a^
grapefruit	4.08	1.03	4.00	1.00	5.00	3.50	1.22	4.00	1.00	5.00	0.0009 ^a^

^a^ U Mann–Whitney test.

**Table 4:** Sweet taste food and beverage preference depending on sweet taste sensitivity score category.

Sweet Taste Sensitivity Category (Score)	Sweet Taste Food and Beverage Preference (Median with Interquartile Range)						*p*-Value
	*n*	All	*n*	Diabetes Group	*n*	Control Group	
incorrect (score 1)	1	4.00 (4.00–4.00)	0	-	1	4.00 (4.00–4.00)	-
average (score 2)	18	4.18 (3.84–4.47)	4	4.00 (3.61–4.34)	14	4.24 (3.86–4.47)	0.4257 ^a^
correct (score 3)	95	4.21 (3.84–4.47)	41	4.00 (3.68–4.37)	54	4.26 (3.95–4.47)	0.0150 ^a^
complete (score 4)	88	4.08 (3.71–4.32)	57	4.05 (3.63–4.26)	31	4.16 (3.89–4.37)	0.1453 ^a^

^a^ U Mann–Whitney test.

**Table 5:** Bitter taste food and beverage preference depending on bitter taste sensitivity score category.

Bitter Taste Sensitivity Category	Bitter Taste Food and Beverage Preference (Median with Interquartile Range)						*p*-Value
	*n*	All	*n*	Diabetes Group	*n*	Control Group	
none (score 0)	5	2.71 (2.29–3.71)	4	3.00 (2.14–3.79)	1	2.71 (2.71–2.71)	-
incorrect (score 1)	19	3.05 (2.29–3.86)	11	3.43 (2.71–3.86)	8	2.29 (1.93–3.50)	0.1253 ^a^
average (score 2)	42	2.91 (2.43–3.57)	18	3.14 (2.57–3.71)	24	2.86 (1.86–3.43	0.2783 ^a^
correct (score 3)	70	3.30 (2.71–3.86)	40	3.71 (3.21–4.14)	30	3.00 (2.14–3.57)	0.0001 ^a^
complete (score 4)	66	3.34 (2.71–4.00)	29	3.86 (3.43–4.29)	37	3.00 (2.29–3.57)	<0.0001 ^a^

^a^ U Mann–Whitney test.

## Data Availability

The raw data supporting the conclusions of this article will be made available by the authors on request.

## Supplementary Materials

The following supporting information can be downloaded at: https://www.mdpi.com/article/10.3390/nu17233752/s1.

## Abbreviations

The following abbreviations are used in this manuscript:

T1DM	Type 1 diabetes
T2DM	Type 2 diabetes
