# Supplementary material for: Food Preferences as a Positive Outcome for Adolescents with Type 1 Diabetes
Source: Nutrients. 2025 Nov 28;17(23):3752. doi: 10.3390/nu17233752 (PMC12694448; doi:10.3390/nu17233752)
Supplement: Supplementary file 1 [file nutrients-17-03752-s001.zip › nutrients-3923725-supplementary.pdf]

Supplementary Materials :

The Figures S1, S2, and S3 show detailed results of sour, salty, and bitter taste recognition by the diabetic group and the control group. Tables S1 and S2 present the results of preferences for salty and fatty foods and beverages in the study groups.

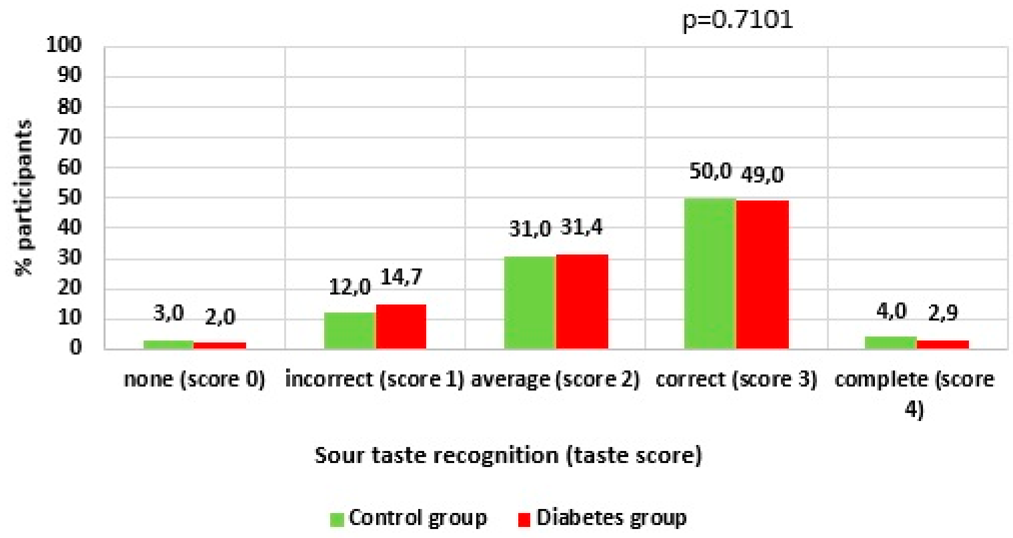

Figure S1. The overall evaluation of the recognition of sour taste.

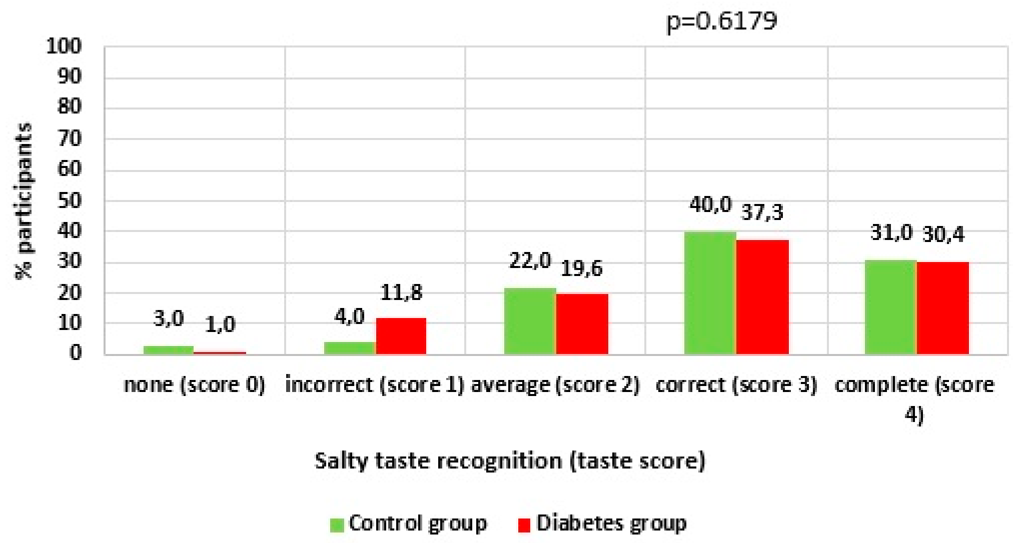

Figure S2. The overall evaluation of the recognition of salty taste.

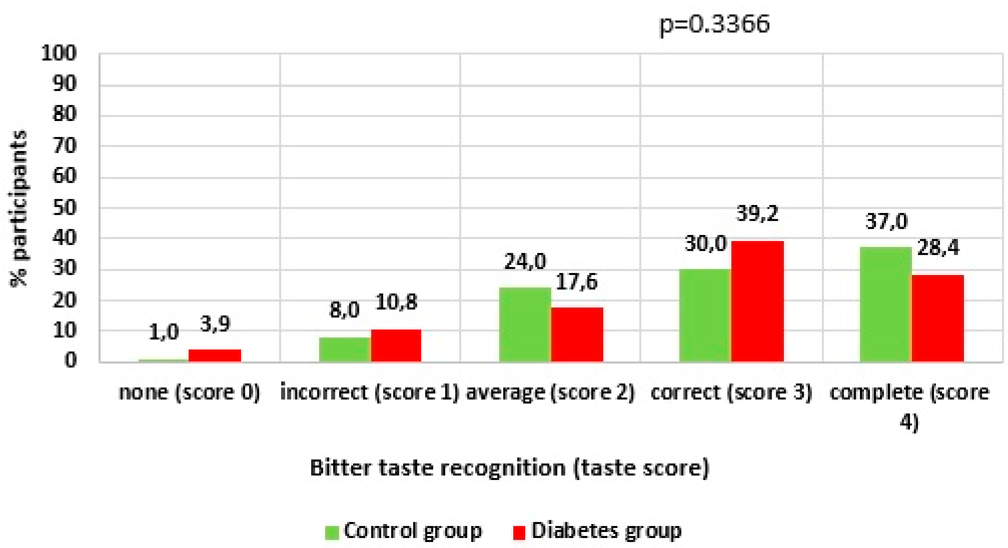

Figure S3. The overall evaluation of the recognition of bitter taste.

Table S1. Salty food and beverage taste preference score

	Diabetes group					Control group					p -Value
	Mean	SD	Median	Min.	Max.	Mean	SD	Median	Min.	Max.	
salty preference score	3.98	0.78	4.13	1.50	5.00	4.19	0.71	4.25	2.25	5.00	0.0442[a]
salted peanuts	3.87	1.02	4.00	1.00	5.00	4.08	0.87	4.00	1.00	5.00	0.2037[a]
salted sticks	4.12	0.90	4.00	2.00	5.00	4.31	0.80	4.00	1.00	5.00	0.1606[a]
crackers	3.82	1.05	4.00	1.00	5.00	4.22	0.85	4.00	1.00	5.00	0.0084[a]
pistachios	4.09	0.97	4.00	1.00	5.00	4.16	0.85	4.00	1.00	5.00	0.7868[a]

[a]U Mann–Whitney test.

Table S2. Fatty food and beverage taste preference score

	Diabetes group					Control group					p -Value
	Mean	SD	Median	Min.	Max.	Mean	SD	Median	Min.	Max.	
fat preference score	3.96	0.66	4.00	2.18	4.91	3.84	0.56	4.00	0.00	4.91	0.0629[a]
mayonnaise	3.06	1.41	3.00	1.00	5.00	2.47	1.30	3.00	1.00	5.00	0.0032[a]
milk	3.19	1.19	3.00	1.00	5.00	3.48	1.16	3.00	1.00	5.00	0.1292[a]
mashed potatoes	4.29	0.56	4.00	2.00	5.00	4.20	0.72	4.00	3.00	5.00	0.5251[a]
hamburger	4.11	1.07	4.00	1.00	5.00	3.92	0.98	4.00	1.00	5.00	0.0710[a]
fried chicken	3.93	1.14	4.00	1.00	5.00	3.85	1.21	4.00	1.00	5.00	0.7479[a]
sasuages	3.71	1.18	4.00	1.00	5.00	3.49	1.12	4.00	1.00	5.00	0.1466[a]
hot dog	4.32	0.92	5.00	1.00	5.00	4.14	1.11	5.00	1.00	5.00	0.3504[a]
salami	3.79	1.04	4.00	1.00	5.00	3.57	1.07	4.00	1.00	5.00	0.1646[a]
fries	4.81	0.42	5.00	4.00	5.00	4.69	0.47	5.00	3.00	5.00	0.1152[a]

chips	4.51	0.66	5.00	2.00	5.00	4.30	0.70	5.00	2.00	5.00	0.0442[a]
kebab	4.32	0.90	5.00	1.00	5.00	4.08	0.95	5.00	1.00	5.00	0.0406[a]

[a]U Mann–Whitney test.
